# Supplementary material for: Photocatalytic Degradation of Ethiofencarb by a Visible Light-Driven SnIn4S8 Photocatalyst
Source: Nanomaterials (Basel). 2021 May 18;11(5):1325. doi: 10.3390/nano11051325 (PMC8157292; doi:10.3390/nano11051325)
Supplement: Supplementary file 1 [file nanomaterials-11-01325-s001.zip › nanomaterials-1135097-supplementary.pdf]

Supplementary Materials

Photocatalytic Degradation of Ethiofencarb by a Visible Light-Driven $SnIn_4S_8$ Photocatalyst

Chiing-Chang Chen [1], Janah Shaya [2,3,4], Kyriaki Polychronopoulou [4,5], Vladimir B. Golovko [6], Siriluck Tesana [6], Syuan-Yun Wang [7] and Chung-Shin Lu [8,*]

[1] Department of Science Education and Application, National Taichung University of Education, Taichung 403, Taiwan; ccchen@mail.ntcu.edu.tw
[2] College of Medicine and Health Sciences, Khalifa University, Abu Dhabi P.O. Box 127788, United Arab Emirates; shaya.janah@ku.ac.ae
[3] College of Arts and Sciences, Khalifa University, Abu Dhabi P.O. Box 127788, United Arab Emirates
[4] Center for Catalysis and Separation, Khalifa University of Science and Technology, Abu Dhabi P.O. Box 127788, United Arab Emirates; kyriaki.polychrono@ku.ac.ae
[5] Department of Mechanical Engineering, Khalifa University of Science and Technology, Abu Dhabi P.O. Box 127788, United Arab Emirates
[6] Department of Chemistry, The MacDiarmid Institute for Advanced Materials and Nanotechnology, University of Canterbury, Christchurch 8140, New Zealand; vladimir.golovko@canterbury.ac.nz (V.B.G.); ri30033ta@gmail.com (S.T.)
[7] School of Medical Applied Chemistry, Chung Shan Medical University, Taichung 402, Taiwan; cindy20122224@gmail.com
[8] Department of General Education, National Taichung University of Science and Technology, Taichung 403, Taiwan
* Correspondence: cslu6@nutc.edu.tw; Tel.: +886-4-2219-6999

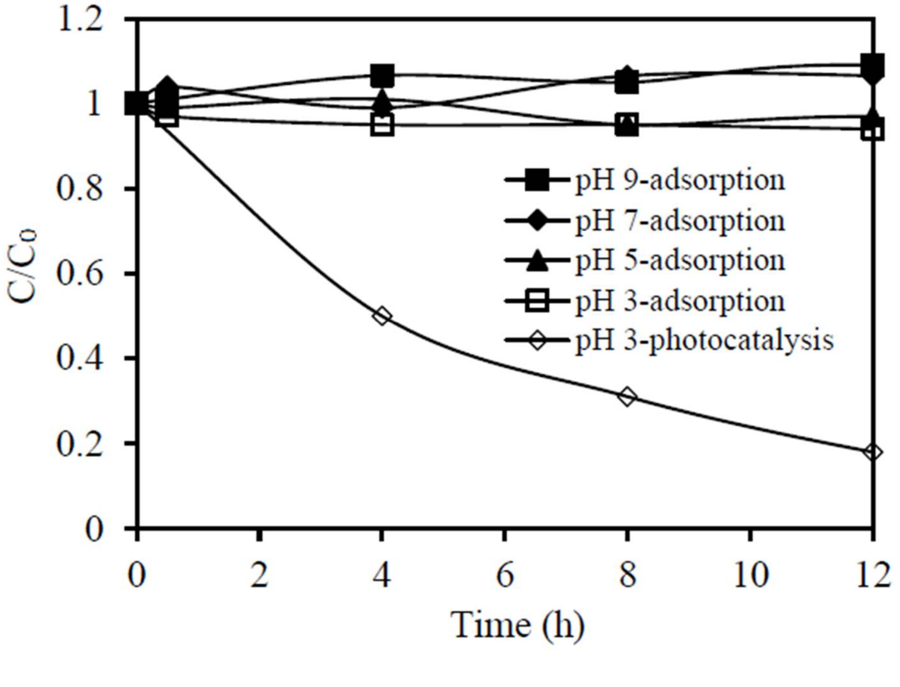

Figure S1. Adsorption of ethiofencarb on $SnIn_4S_8$ at different pH conditions. Experimental conditions: ethiofencarb concentration 10 mg L^{-1}; $SnIn_4S_8$ concentration 0.5 g L^{-1}.

Compound **1**

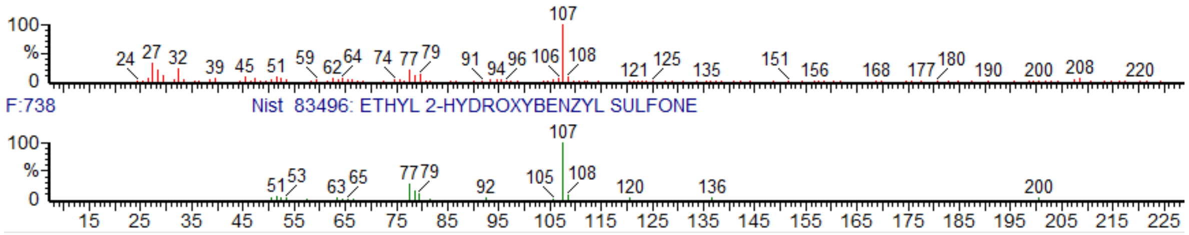

Compound **2**

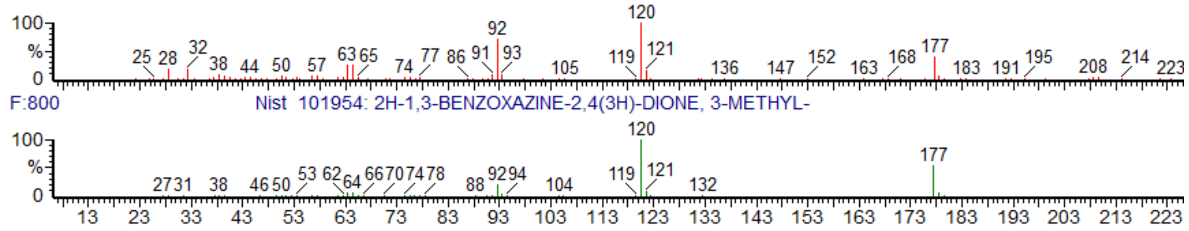

Compound **3**

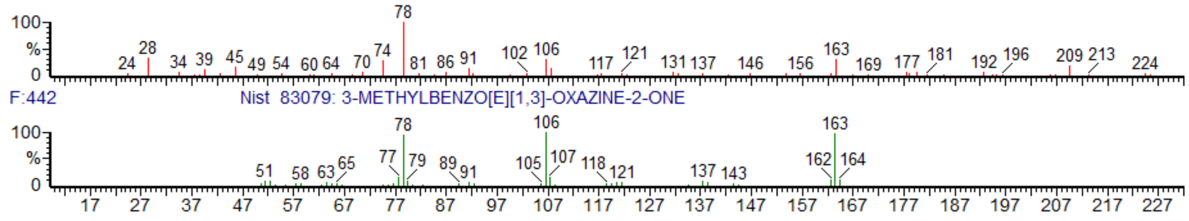

Compound **4**

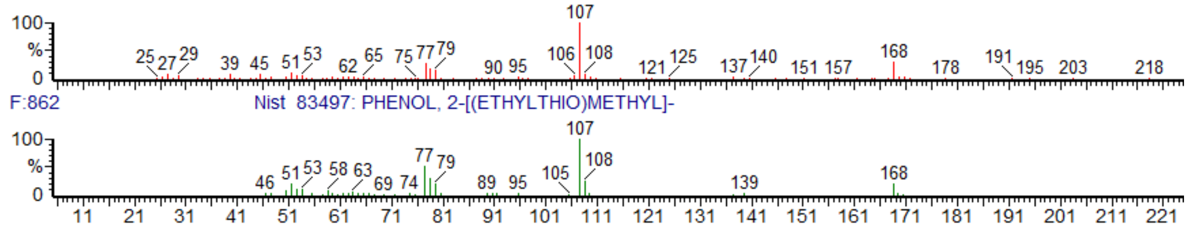

Compound **5**

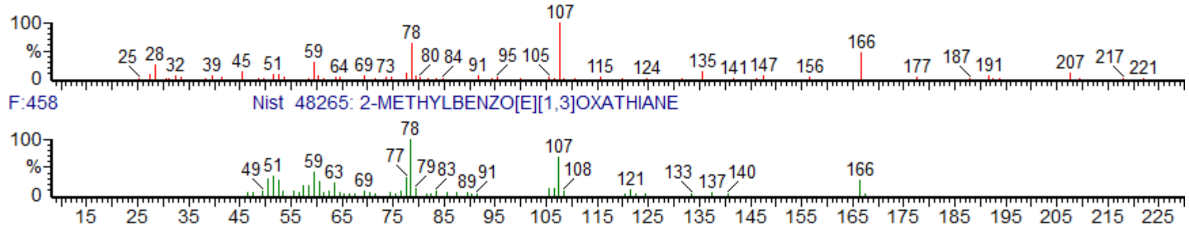

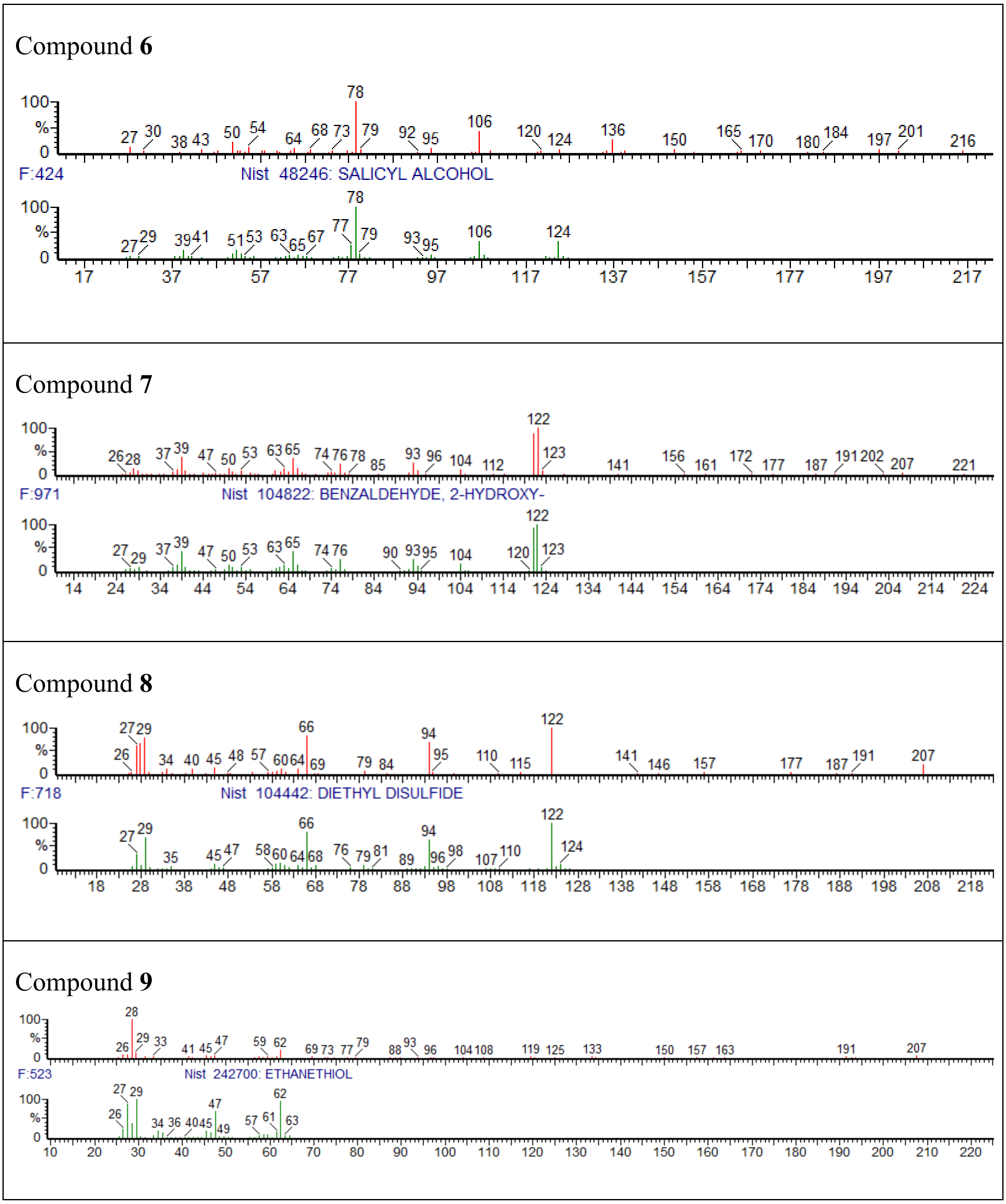

Figure S2. Mass spectra of intermediates formed during the photodegradation of ethiofencarb after they were separated by GC/MS method.

Table S1. Characteristics of the real water sample.

Parameter	River water	Lake water
pH	6.30	6.24
Conductivity (μmho cm^{-1})	420	378
Turbidity (NTU)	2.9	4.9
TOC (mg L^{-1})	13.43	3.44
Sulfate (mg L^{-1})	48.1	50.9
Chloride (mg L^{-1})	22.6	16.7
Nitrate (mg L^{-1})	84.8	2.9
Nitrite (mg L^{-1})	1.12	0.02
